# Selective efferent vagal stimulation in heart failure

**DOI:** 10.1113/EP090866

**Published:** 2023-09-26

**Authors:** Lindsea C. Booth, Baagavi Saseetharan, Clive N. May, Song T. Yao

**Affiliations:** ^1^ Florey Institute of Neuroscience and Mental Health University of Melbourne Parkville VIC Australia; ^2^ Department of Anatomy and Physiology The University of Melbourne Melbourne Australia

**Keywords:** heart failure, optogenetic nerve stimulation, vagal nerve stimulation

## Abstract

Patients diagnosed with heart failure have high rates of mortality and morbidity. Based on promising preclinical studies, vagal nerve stimulation has been trialled in these patients using whole nerve electrical stimulation, but the results have been mixed. This is, at least in part, due to an inability to selectively recruit the activity of specific fibres within the vagus with whole nerve electrical stimulation, as well as not knowing which the ‘therapeutic’ fibres are. This symposium review focuses on a population of cardiac‐projecting efferent vagal fibres with cell bodies located within the dorsal motor nucleus of the vagus nerve and a new method of selectively targeting these projections as a potential treatment in heart failure.

## Abstract (highlights)

- **What is the topic of this review?**
  Selective efferent vagal stimulation in heart failure.
- **What advances does it highlight?**
  Selectively targeting a population of cardiac‐projecting efferent vagal fibres with cell bodies within the dorsal motor nucleus of the vagus using optogenetics slows the progression of heart failure in rats.

## 1 INTRODUCTION

Heart failure patients have an impaired quality of life, experiencing dyspnoea, fatigue, oedema, sleeping difficulties, depression and chest pain, with a high risk of hospital admission and a 5‐year mortality rate of 75% (Levy et al., 2002). Although the number of patients hospitalised with heart failure has declined in the last few decades (Chen et al., 2011), mortality and morbidity remain high and new treatment options are required.

Based on promising experimental animal studies, vagus nerve stimulation (VNS) has been trialled in patients with heart failure using whole nerve electrical stimulation. The results have been mixed, with high rates of side effects reported. These are undoubtably in part due to the non‐specific nature of whole nerve electrical stimulation and a lack of clear understanding of which vagal fibres need to be recruited to confer benefit in heart failure. This symposium review will focus on a population of vagal fibres which originate in the dorsal motor nucleus of the vagus (DMV), their functional role in the control of cardiac contractility and a novel optogenetic method for selectively targeting these fibres as a potential treatment of heart failure.

## 2 VAGAL STIMULATION IN HEART FAILURE

There is a well‐documented loss of cardiac parasympathetic (vagal) activity in heart failure (Eckberg et al., 1971; Watson et al., 2007) and indicators of decreased vagal tone are strong predictors of arrhythmias and adverse outcomes in heart failure patients (Farrell et al., 1991). In animal models of heart failure, VNS has been shown to improve survival (Li et al., 2004), decrease the number of arrhythmias (Zheng et al., 2005), increase the left ventricular (LV) ejection fraction (Zhang et al., 2009) and improve end‐systolic LV function (Hamann et al., 2013).

In view of these findings, electrical stimulation of the vagus nerve has been trialled in heart failure patients (Dusi & De Ferrari, 2021; van Bilsen et al., 2017). Although in early studies LV end systolic volume decreased (Schwartz et al., 2008), the results obtained in randomised controlled trials were less convincing, showing moderate improvements in the 6 min walk test and quality of life scores (Gold et al., 2016). Importantly, across the trials, there have been high rates of reported side effects (e.g., cough, neck pain) and a large range of maximally tolerated stimulation currents have been used, raising questions about whether therapeutic levels of stimulation were reached in all studies (Dusi & De Ferrari, 2021; van Bilsen et al., 2017).

The inability to selectively recruit the activity of ‘therapeutic’ cardiac fibres within the vagus nerve remains a major limitation of whole nerve electrical stimulation. Indeed, the high intensity electrical stimulation required to recruit unmyelinated/lightly myelinated efferent fibres (to increase/restore cardiac parasympathetic activity) simultaneously captures afferent fibres (e.g., pulmonary afferents that inhibit respiration and Aδ fibres that cause cough), leading to significant side effects (Dusi & De Ferrari, 2021). Without precise targeting of specific vagal fibres, it is unsurprising that whole nerve electrical stimulation has had such mixed results in the clinical trials.

3

The vagus nerve innervates virtually all thoracic and abdominal visceral organs and is a mixed nerve containing efferent (20%) and afferent (80%) fibres (for an extensive review see Ottaviani & Macefield, 2022). On a basic level, afferent vagal fibres carry sensory information from the internal organs to the central nervous system and efferent vagal fibres carry motor signals from the brainstem to visceral organs. However, it should be acknowledged that these fibres exist within a highly complex system of cardiac innervation, and the neuromodulation of different targets within this system has been recently reviewed by Herring et al. (2019). Despite a growing understanding of the vagus nerve and its components, it remains unclear exactly which vagal fibres need to be stimulated to confer the most benefit in heart failure, with a number of potential candidates. For example, in rats, stimulation of vagal afferent fibres has been shown to reset the arterial baroreflex neural arc and inhibit splanchnic sympathetic activity (Saku et al., 2014). On the other side, efferent vagal fibres regulate heart rate, contractility and excitability (Hsieh et al., 1998). When stimulated, the released acetylcholine activates muscarinic receptors in the heart, decreasing heart rate and atrial and ventricular contractility. Hence stimulation of efferent fibres may reduce the cardiac workload and oxygen consumption. Vagal efferent stimulation has also been shown to reduce free radical generation (Tsutsumi et al., 2008) and attenuate noradrenaline release by cardiac sympathetic nerves (Levy & Blattberg, 1976). In addition, electrical stimulation of the vagus nerve has a powerful anti‐inflammatory effect (Komegae et al., 2018), which is likely to be beneficial in heart failure.

Although recruitment of afferent fibres may have beneficial effects in heart failure, the significant side effects of whole nerve stimulation are primarily driven by afferent fibre activation. Therefore, efferent vagal fibres have been the primary target of selective vagal stimulation (discussed below). Vagal efferent fibres originate from the dorsal motor nucleus of the vagus (DMV) and the nucleus ambiguus in the medulla oblongata (Dampney, 1981). Neurons in the nucleus ambiguus provide the majority of the efferent motor innervation to the cardiac ganglia and modulate heart rate (Hsieh et al., 1998). The DMV neurons provide efferent motor output to ganglia innervating the thoracic and abdominal viscera, with a small proportion innervating the cardiac ganglia (Hsieh et al., 1998). This lesser‐studied, but important, population of DMV neurons projecting to the heart will be discussed in more depth below.

## 4 SELECTIVELY TARGETING THE EFFERENT FIBRES OF THE VAGUS NERVE

There has been a recent flurry of activity around increasing selectivity of vagal nerve stimulation, with the advent of broader neuroscience‐based bioelectric neuromodulation and a greater understanding of the organisation of the vagus nerve in large animals and humans (e.g., Jayaprakash et al., 2023; Settell et al., 2020; Thompson et al., 2023). A comprehensive assessment of these techniques is beyond the scope of this symposium review; however, they include spatially selective VNS (Aristovich et al., 2021), directionally selective VNS (Villalobos et al., 2023), anatomically selective VNS (Butt et al., 2020) and targeted VNS using optogenetics, which is the focus of this review.

5

In 2015, Machhada et al. (2015) demonstrated that silencing neurons in the DMV, including those projecting to the heart, led to shortening of the ventricular effective refractory period and, importantly, lowered the threshold for triggered ventricular tachycardia in anaesthetised rats. These effects on ventricular excitability were shown to be mediated by nitric oxide (Machhada et al., 2015), in line with the evidence obtained using an isolated innervated rabbit heart model (Brack et al., 2011, 2009, 2021, 2009). Therefore, targeted activation of these vagal efferents in chronic heart failure has the potential to reduce susceptibility to ventricular arrhythmias. The same group then went on to show, in anaesthetised rats, that the cardiac projecting DMV neurons exert a tonic inhibitory drive of left ventricular contractility (Machhada et al., 2016).

The effects of stimulating this population of neurons in heart failure were determined in a more recent study, where the DMV fibres were selectively stimulated using optogenetic approach in a rodent model of myocardial infarction‐induced heart failure (Machhada et al., 2020). DMV neurons express the transcription factor Phox2 and were targeted to express a light‐sensitive chimeric channelrhodopsin derivative, ChIEF, fused with a fluorescent protein, tdTomato. Optogenetic stimulation of DMV neurones in the first 4 weeks after the myocardial infarction was sufficient to slow the progression of heart failure and increase exercise capacity (Machhada et al., 2020). Interestingly, in addition to improvements seen in rats with heart failure, quantitatively similar functional improvements were also seen in the control (sham‐myocardial infarction) group. This control group is rarely, if ever, included in VNS experiments of this type. The results obtained in the experiments using optogenetic stimulation of the vagal efferent fibres suggest that the mechanisms driving the beneficial effects of VNS in heart failure are gain‐of‐function.

The mechanisms underlying these beneficial changes in rats with heart failure have not been fully elucidated; however, in heart failure, increased activity of the sympathetic nervous system leads to a detrimental downregulation and desensitisation of myocardial β_1_‐adrenoreceptors (β‐AR) and upregulation of a key negative regulator of β‐AR‐mediated signalling, G‐protein coupled receptor kinase 2 (GRK2) (Woo & Xiao, 2012). In a separate study, Machhada et al. have demonstrated that selective optogenetic activation of the DMV in healthy rats decreases the expression of GRK2, which increases responsiveness of the ventricular myocardium to β_1_‐adrenergic stimulation (Machhada et al., 2017). Therefore, this is one pathway through which optogenetic stimulation of efferent fibres could slow the progression of heart failure (Machhada et al., 2020). The benefits of inhibiting overactive GRKs in heart failure has been recently reviewed (Pfleger et al., 2019).

The beneficial effects of efferent vagal fibre stimulation in heart failure may not all be due to specific activation of cardiac projections. The caudal region of the DMV has been shown to be the origin of cardiac efferent fibres in rats (Machhada et al., 2016); however, there is overlap with neurons projecting to other regions, including the abdominal viscera. This observation raises the question of whether there are additional cardioprotective mechanisms recruited by activation of efferent vagus nerve projections to other organs. For example, it was shown that the vagally mediated release of the gut hormone GLP‐1 is cardioprotective (Basalay et al., 2016). In addition, there is a well‐recognised, though poorly understood, role for inflammation in the progression of heart failure (Hanna & Frangogiannis, 2020) and it is established that vagal nerve stimulation has anti‐inflammatory actions (Caravaca et al., 2022). For example, there is evidence that in a pacing‐induced canine model of heart failure, vagal stimulation reduced the inflammatory marker plasma C‐reactive protein (Zhang et al., 2009). Therefore, reduced inflammation and increased release of circulating factors, such as GLP‐1, may help slow the progression of heart failure (Machhada et al., 2020). Future studies should focus on determining the exact mechanisms by which optogenetic stimulation of the DMV slows the progression of heart failure in rats following myocardial infarction.

## 6 TARGETING EFFERENT VAGAL FIBRES USING OPTOGENETICS IN A LARGE MAMMAL

**FIGURE 1:**
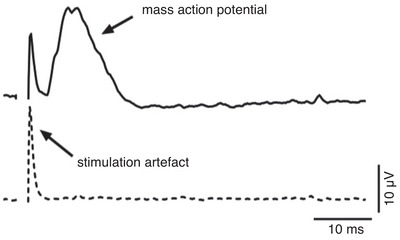
Mass action potential recorded from the transected end of an opsin‐positive cervical vagus nerve, activated with an LED (99 mW/mm^2^, 2 ms, 1 Hz). Distance between LED and recording electrodes ∼70 mm). (Booth et al., 2021).

Several reports have demonstrated the use of optogenetic tools to stimulate peripheral nerves in small animal models (Maimon et al., 2017; Pincus et al., 2020; Rajendran et al., 2019). However, if optogenetic techniques are to be translated to clinical use and the mechanisms of action dissected, the efficacy of stimulating specific fibres needs to be demonstrated in clinically relevant large animal models. We recently published a technique of targeting the DMV in sheep and showed effective optical stimulation of the cervical vagus nerve (Figure 1) (Booth et al., 2021). This site is easily accessible and is used for implantation of electrodes for electrical stimulation of the vagus. Twelve weeks after the injection of the vectors, sheep expressed optogenetic channels in a subset of efferent vagal fibres at the level of the cervical and cardiac vagus. Mass action potentials were recorded in fascicles of the cervical vagus nerve with light intensities of between 5 and 99 mW/mm^2^, with a conduction velocity of approximately 6 m/s (Booth et al., 2021). The conduction velocity indicates that the axons of DMV neuron are lightly myelinated (B fibres) in large animals, which is in line with studies in cats (Jones et al., 1998), and in contrast with data from rats, where the DMV neurons have C fibre axons (Nishimura & Oomura, 1987).

As outlined above, the mechanism by which efferent vagal stimulation is effective in heart failure remains incompletely understood. The large animal model is a powerful tool to dissect the effects and functional significance of efferent vagal innervation of specific organs and to map out critical pathways that are beneficial in heart failure.

## AUTHOR CONTRIBUTIONS

All authors approved the final version of the manuscript and agree to be accountable for all aspects of the work in ensuring that questions related to the accuracy or integrity of any part of the work are appropriately investigated and resolved. All persons designated as authors qualify for authorship, and all those who qualify for authorship are listed.

## CONFLICT OF INTEREST

The authors have no competing interests to declare.
